# Protective Face Mask Filter Capable of Inactivating SARS-CoV-2, and Methicillin-Resistant *Staphylococcus aureus* and *Staphylococcus epidermidis*

**DOI:** 10.3390/polym13020207

**Published:** 2021-01-08

**Authors:** Miguel Martí, Alberto Tuñón-Molina, Finn Lillelund Aachmann, Yukiko Muramoto, Takeshi Noda, Kazuo Takayama, Ángel Serrano-Aroca

**Affiliations:** 1 Biomaterials and Bioengineering Lab, Centro de Investigación Traslacional San Alberto Magno, Universidad Católica de Valencia San Vicente Mártir, c/Guillem de Castro 94, 46001 Valencia, Spain; miguel.marti@ucv.es (M.M.); alberto.tunon@ucv.es (A.T.-M.); 2 The Norwegian Biopolymer Laboratory (NOBIPOL), Department of Biotechnology and Food Science, NTNU Norwegian University of Science and Technology, Sem Sælands vei6-8, N-7491 Trondheim, Norway; finn.l.aachmann@ntnu.no; 3 Laboratory of Ultrastructural Virology, Institute for Frontier Life and Medical Sciences, Kyoto University, Kyoto 606-8507, Japan; muramo@infront.kyoto-u.ac.jp (Y.M.); t-noda@infront.kyoto-u.ac.jp (T.N.); 4 Center for iPS Cell Research and Application, Kyoto University, Kyoto 606-8397, Japan

**Keywords:** SARS-CoV-2, MRSA, MRSE, face mask filter, benzalkonium chloride, COVID-19, multidrug-resistant bacteria

## Abstract

Face masks have globally been accepted to be an effective protective tool to prevent bacterial and viral transmission, especially against indoor aerosol transmission. However, commercial face masks contain filters that are made of materials that are not capable of inactivating either SARS-CoV-2 or multidrug-resistant bacteria. Therefore, symptomatic and asymptomatic individuals can infect other people even if they wear them because some viable viral or bacterial loads can escape from the masks. Furthermore, viral or bacterial contact transmission can occur after touching the mask, which constitutes an increasing source of contaminated biological waste. Additionally, bacterial pathogens contribute to the SARS-CoV-2-mediated pneumonia disease complex, and their resistance to antibiotics in pneumonia treatment is increasing at an alarming rate. In this regard, herein, we report the development of a non-woven face mask filter fabricated with a biofunctional coating of benzalkonium chloride that is capable of inactivating more than 99% of SARS-CoV-2 particles in one minute of contact, and the life-threatening methicillin-resistant *Staphylococcus aureus* and *Staphylococcus epidermidis* (normalized antibacterial *halos* of 0.52 ± 0.04 and 0.72 ± 0.04, respectively). Nonetheless, despite the results obtained, further studies are needed to ensure the safety and correct use of this technology for the mass production and commercialization of this broad-spectrum antimicrobial face mask filter. Our novel protective non-woven face mask filter would be useful for many healthcare workers and researchers working in this urgent and challenging field.

## 1. Introduction

The severe acute respiratory syndrome coronavirus 2 (SARS-CoV-2) was first reported in Wuhan, Hubei province, China, in December 2019 [1]. The rapid spread of this pathogen, which has caused the current COVID-19 pandemic, is putting at high risk the health and economy of the most developed and underdeveloped countries. According to the World Health Organization (WHO), the current COVID-19 outbreak has 83,979,200 global cases and 1,827,796 global deaths in more than 200 countries (data as of 2 January 2021) [2]. SARS-CoV-2 is the third coronavirus causing severe pneumonia [3,4], an infection of the lungs usually caused by bacteria and viruses [5,6]. The death risk of viral pneumonias can increase when co-infection can be caused by viruses in the setting of community-acquired bacterial pneumonia such as the lethal *Streptococcus pneumoniae* [7,8,9,10], with additional symptoms of bacterial pneumonia [11]. New pathogens, such as SARS-CoV-2, which can coexist with a broad range of other types of clinically relevant bacteria, including multidrug-resistant strains, constitute a real-life threat to humans. In addition, antibiotic resistance in bacterial pneumonia treatment is a widespread problem that is increasing at an alarming rate [12,13]. The SARS-CoV-2 pathogen is stable from hours to days in aerosols and surfaces of different chemical natures such as copper, cardboard, plastic, aluminum or stainless steel surfaces, demonstrating that infections can be easily transmitted through the air via microdroplets or direct contact after touching contaminated surfaces [14,15,16,17,18]. This coronavirus can spread faster than its two ancestors SARS-CoV and MERS-CoV [19] through coughing, sneezing, touching or breathing [20], and more broadly through asymptomatic carriers [21,22,23]. Recent studies have demonstrated that direct indoor aerosol transmission or ventilation systems can potentially transmit SARS-CoV-2 [24,25,26]. Although the confinements conducted in many countries flattened the epidemic curve before the hot season [27,28], SARS-CoV-2 continues to spread globally.

SARS-CoV-2 is an enveloped, positive-sense, single-stranded RNA virus [29] that belongs to Baltimore group IV [30]. Other enveloped RNA viruses such as influenza A (H1N1) can be inactivated by quaternary ammonium compounds such as benzalkonium chloride (BAK) [31]. It has been recently remarked, however, that further evaluation of the effectiveness of BAK against coronaviruses is needed [32] because the Centers for Disease Control and Prevention have reported that available evidence indicates BAK has less reliable activity against certain bacteria and viruses than either of the alcohols [33]. However, a recent report has shown the in vitro virucidal activity of ethanol (70%), povidone–iodine (7.5%), chloroxylenol (0.05%), chlorhexidine (0.05%) or benzalkonium chloride (0.1%) was similar when used as disinfectants against SARS-CoV-2 [18]. Thus, the oral rinse Dequonal, which contains BAK, has shown virucidal activity against SARS-CoV-2 under conditions mimicking nasopharyngeal secretions to support the idea that oral rinsing might reduce the viral load of saliva and could thus lower the transmission of SARS-CoV-2 [34]. Furthermore, very recently, a preprint reported an oil-in-water nanoemulsion formulation containing 0.13% BAK that has demonstrated safe and broad antiviral activity against enveloped viruses such as SARS-CoV-2, human coronavirus, respiratory syncytial virus and influenza B [35]. In that study, the repeated application of this BAK-containing nanoemulsion, twice daily for 2 weeks onto rabbit nostrils indicated safety with no irritation. In fact, this chemical compound is widely used as a disinfectant against bacteria, viruses, pathogenic fungi and mycobacteria, and it has been approved by the Food and Drug Administration as a skin disinfectant [36].

Face masks have been accepted as effective protective tools by blocking the pass of viral and bacterial particles [37]. However, if the filters that contain the face masks are made of composite materials with antimicrobial activity, the protection of these tools could increase even more. Thus, several antiviral face mask materials against SARS-CoV-2 have been recently proposed. However, all of these studies, some of them not peer-reviewed yet, propose expensive materials such as graphene [38], copper [39] or silver [40]. These antiviral composites are produced with complex and costly manufacturing processes, which render them non-viable for a global solution of the current COVID-19 pandemic strongly affecting both developed and underdeveloped countries. In this regard, we hypothesize here that the physical adsorption of BAK via the dip coating method [41] onto the surface of a commercial non-woven fabric filter, which is commonly used in the production of face masks in the present pandemic, could produce a low-cost antiviral filter that could inhibit the infection capacity of SARS-CoV-2. Non-woven filters are lightweight, flexible, resilient, provide good bacteria filtration and air permeability, are cost-effective materials for masks, have a lower manufacturing cost and are hygienic and clean as they are for single use [42]. Furthermore, due to the previously reported antibacterial activity of BAK against Gram-positive bacteria [43], we also expect that the developed BAK filter will be able to inhibit the bacterial growth of two clinically relevant multidrug-resistant bacteria: methicillin-resistant *Staphylococcus aureus* (MRSA) and *Staphylococcus epidermidis* (MRSE). In addition to the current COVID-19 pandemic, antibiotic resistance is another increasing challenge of the present century. According to the World Health Organization (WHO), antibiotic resistance will be one of the leading causes of death over other important diseases such as cancer by the year 2050 [44]. In fact, MRSE is a nosocomial pathogen that is spreading globally and is often the cause of catheter-associated disease, especially among low-birth-weight premature infants [45,46]. MRSA is causing global health problems, especially in medical instruments and catheters because *S. aureus* is a human pathogen that can easily develop resistance to antibiotics [47,48]. Therefore, in this study, we attempted to develop a low-cost protective face mask filter capable of inactivating SARS-CoV-2, and MRSA and MRSE.

## 2. Materials and Methods

### 2.1. Dip Coating of Commercial Face Filter Masks

Disks specimens of approximately 10 mm in diameter were prepared with a non-woven spunlace fabric filter (commercial filters used for face masks, NV EVOLUTIA, Valencia, Spain) by dry-cutting with a cylindrical punch. Face mask filter (BAK filter) disks (*n* = 6) were produced by the dip coating method [41] using commercial 70% ethyl alcohol with 0.1% *w*/*w* benzalkonium chloride (Montplet, Barcelona, Spain) for 1 min at 25 °C to achieve a dry BAK content, determined gravimetrically, of 0.46 ± 0.13% *w*/*w*. Another face mask filter (S filter) disk (*n* = 6) was subjected to the same dip coating treatment but using only an absolute ethanol/distilled water solution (70/30% *v*/*v*) without BAK for 1 min at 25 °C. Untreated face mask filter (U filter) disks (*n* = 6) were produced as reference material. The disks were subsequently dried at 60 °C for 48 h to constant weight and sterilized by UV radiation (TELSTAR Technologies S.L, Terrassa, Spain) for one hour per each side.

### 2.2. Characterization of the Benzalkonium Chloride

Nuclear magnetic resonance (NMR) was applied for the characterization of the benzalkonium chloride used in the biofunctional coating of the commercial non-woven filter. Prior to NMR sample preparation, the ethanol/water solvent was evaporated from commercial Montplet 70% ethyl alcohol with benzalkonium chloride (99.9/0.1% *w*/*w*) at 25 °C. After that, the sample of benzalkonium chloride was prepared by dissolving 10 mg in 550 µL D_2_O (D, 99.9%) (Sigma-Aldrich, City Norway) and transferred to a 5 mm LabScape Stream NMR tube. The NMR experiments were recorded on a BRUKER AVIIIHD 800 MHz (Bruker BioSpin AG, Fälladen, Switzerland) equipped with a 5mm cryogenic CP-TCI. All NMR recording was performed at 25 °C or 37 °C. For the characterization of benzalkonium chloride the following spectra were recorded: 1D proton, 2D double quantum filtered correlation spectroscopy (DQF-COSY) and 2D ^13^C heteronuclear single quantum coherence (HSQC) with multiplicity editing. TMS was used as a chemical shift reference for proton and carbon chemical shifts. The spectra were recorded, processed and analyzed using TopSpin 3.7 software (Bruker BioSpin AG, Fällanden, Switzerland).

### 2.3. Electron Microscopy

A Zeiss Ultra 55 field emission scanning electron microscope (FESEM, Carl Zeiss Microscopy, Jena, Germany) was operated at an accelerating voltage of 3 kV to observe the porous morphology of the treated and untreated non-woven face mask filters at a magnification of ×100 and ×1000. The filter samples were prepared to be conductive by platinum coating with a sputter coating unit.

### 2.4. Phage Phi 6 Host Culture

*Pseudomonas syringae* (DSM 21482) from the Leibniz Institute DSMZ–German Collection of Microorganisms and Cell cultures GmbH (Braunschweig, Germany) was cultured in solid tryptic soy agar (TSA, Liofilchem) and subsequently in liquid tryptic soy broth (TSB, Liofilchem). Liquid incubation was carried out at 25 °C and 120 rpm.

### 2.5. Phage Phi 6 Propagation

Phage phi 6 (DSM 21518) propagation was carried out according to the specifications provided by the Leibniz Institute DSMZ–German Collection of Microorganisms and Cell Cultures GmbH (Braunschweig, Germany).

### 2.6. Antiviral Test Using the Biosafe Viral Model

A volume of 50 μL of a phage suspension in TSB was added to each filter at a titer of about 1 × 10^6^ plaque-forming units per mL (PFU/mL) and allowed to incubate for 1, 10 and 30 min. Each filter was placed in a falcon tube with 10 mL TSB and sonicated for 5 min at 24 °C. After that, each tube was vortexed for 1 min. Serial dilutions of each falcon sample were made for phage titration, and 100 μL of each phage dilution was contacted with 100 μL of the host strain at OD_600nm_ = 0.5. The infective capacity of the phage was measured based on the double-layer method [49], where 4 mL of top agar (TSB + 0.75% bacteriological agar, Scharlau) and 5 mM CaCl_2_ were added to the phage–bacteria mixture which was poured on TSA plates. The plates were incubated for 24–48 h in an oven at 25 °C. The phage titer of each type of sample was calculated in PFU/mL and compared with the control, that is, 50 μL of phage added to the bacteria without being in contact with any filter and without being sonicated. The antiviral activity in log reductions of titers was estimated at 1, 10 and 30 min of contact with the virus model. It was checked that the residual amounts of disinfectants in the titrated samples did not interfere with the titration process and the sonication–vortex treatment did not affect the infectious capacity of the phage. The antiviral tests were performed three times during two different days (*n* = 6) to ensure reproducibility.

### 2.7. Antiviral Tests Using SARS-CoV-2

The SARS-CoV-2 strain used in this study (SARS-CoV-2/Hu/DP/Kng/19-027) was kindly gifted to us by Dr. Tomohiko Takasaki and Dr. Jun-Ichi Sakuragi at the Kanagawa Prefectural Institute of Public Health. The virus was plaque-purified and propagated in Vero cells. SARS-CoV-2 was stored at −80 °C.

A volume of 50 μL of a virus suspension in phosphate-buffered saline (PBS) was added to each filter at a titer dose of 1.3 × 10^5^ TCID50/filter, and then incubated for 1 min at room temperature. Then, 1 mL PBS was added to each filter, and then vortexed for 5 min. After that, each tube was vortexed for 5 min at room temperature.

Viral titers were determined through median tissue culture infectious dose (TCID50) assays inside a Biosafety Level 3 laboratory at Kyoto University. Briefly, TMPRSS2/Vero cells [50] (JCRB1818, JCRB Cell Bank), cultured with the minimum essential media (MEM, Sigma-Aldrich) supplemented with 5% fetal bovine serum (FBS), 1% penicillin/streptomycin, were seeded into 96-well plates (Thermo Fisher Scientific). Samples were serially diluted 10-fold from 10^−1^ to 10^−8^ in the culture medium. Dilutions were placed onto the TMPRSS2/Vero cells in triplicate and incubated at 37 °C for 96 h. Cytopathic effect was evaluated under a microscope. TCID50/mL was calculated using the Reed–Muench method.

### 2.8. Antibacterial Tests

The agar disk diffusion tests were performed to analyze the antibacterial activity of the treated and untreated filters [51,52]. Lawns of methicillin-resistant *Staphylococcus aureus*, COL [53], and the methicillin-resistant *Staphylococcus epidermidis*, RP62A [54], in a concentration of about 1.5 × 10^8^ CFU/mL in tryptic soy broth, were cultivated on trypticase soy agar plates. The sterilized disks were placed upon the lawns of bacteria to be incubated aerobically at 37 °C for 24 h. The antibacterial activity of the tested filter disks was expressed according to Equation (1) [51]:

(1) $$nw_{halo}=\frac{\frac{d_{iz}-d}{2}}{d}$$

where *nw_halo_* indicates the normalized width of the antimicrobial inhibition zone, *d_iz_* is the inhibition zone diameter and *d* refers to the sample disk diameter. These diameters were measured by image software analysis (Image J, Wayne Rasband (NIH), Bethesda, MD, USA). The tests were performed six times on different days to ensure reproducibility.

### 2.9. Statistical Analysis

The statistical analyses were performed by ANOVA followed by Tukey’s post hoc test (* *p* > 0.05, *** *p* > 0.001) on GraphPad Prism 6 software (gGraphPad Software Inc., San Diego, CA, USA).

## 3. Results

### 3.1. Nuclear Magnetic Resonance of Benzalkonium Chloride

**Figure 1:**
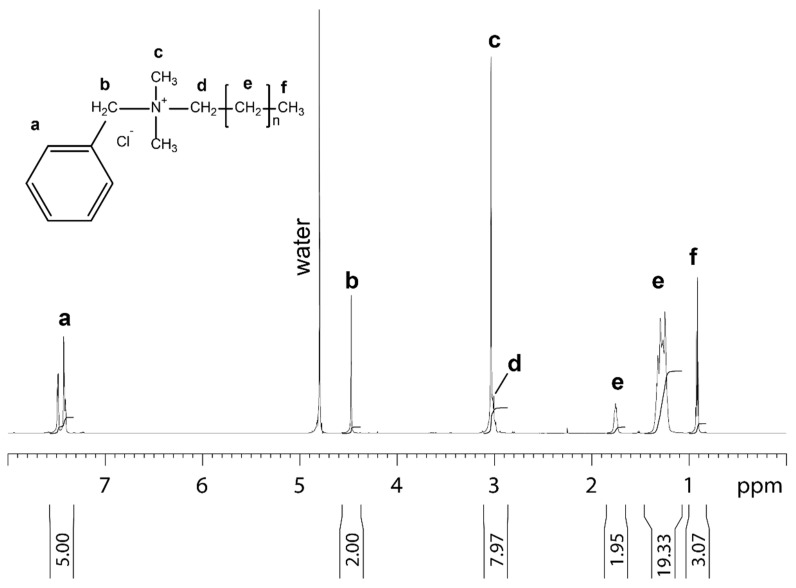
1D proton NMR spectrum of benzalkonium chloride dissolved in 99.9% D_2_O recorded at 25 °C. Molecular structure, assignment and integral for benzalkonium chloride are shown. The letters at the molecular structure and the spectrum indicate the proton in the different chemical subgroups of benzalkonium chloride.

The benzalkonium chloride used in this study for the treatment of the non-woven filter analyzed by NMR is shown in Figure 1.

### 3.2. Porous Morphology of the Non-Woven Face Mask Filters

**Figure 2:**
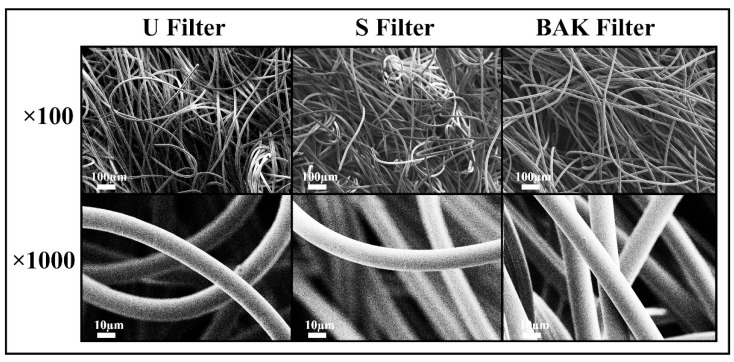
Morphology of the non-woven face mask filters by field emission scanning electron microscopy. Untreated filter (U filter), filter treated by dip coating with the ethanol-based solvent (S filter) and filter with 0.46 ± 0.13% *w*/*w* of biofunctional benzalkonium chloride (BAK) coating (BAK filter) at two magnifications (×100 and ×1000).

The porous morphology images of the commercial and treated non-woven face mask filter are shown in Figure 2.

FESEM observation showed no signs of porous morphological change after performing the dip coating with both ethanol-based solvent or the BAK compound. These results suggest no change of breathability or bacterial filtration efficiency required for their commercialization according to the European standard for community face coverings (CWA 17553:2020).

### 3.3. Antiviral Tests with Phage Phi 6 and SARS-CoV-2

**Figure 3:**
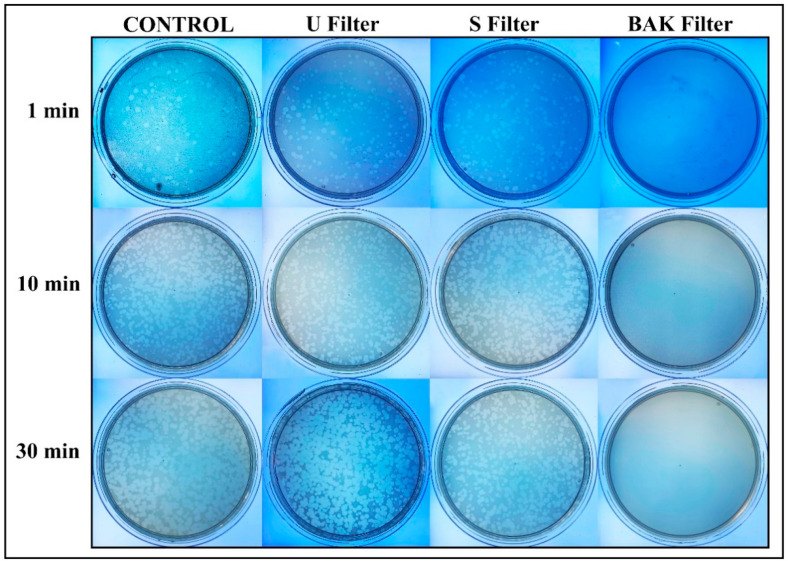
Loss of phage phi 6 viability measured by the double-layer method. Phage 6 titration images of undiluted samples for control, untreated filter (U filter), filter treated by dip coating with the ethanol-based solvent (S filter) and filter with the biofunctional BAK coating (BAK filter) at 1, 10 and 30 min of viral contact.

**Figure 4:**
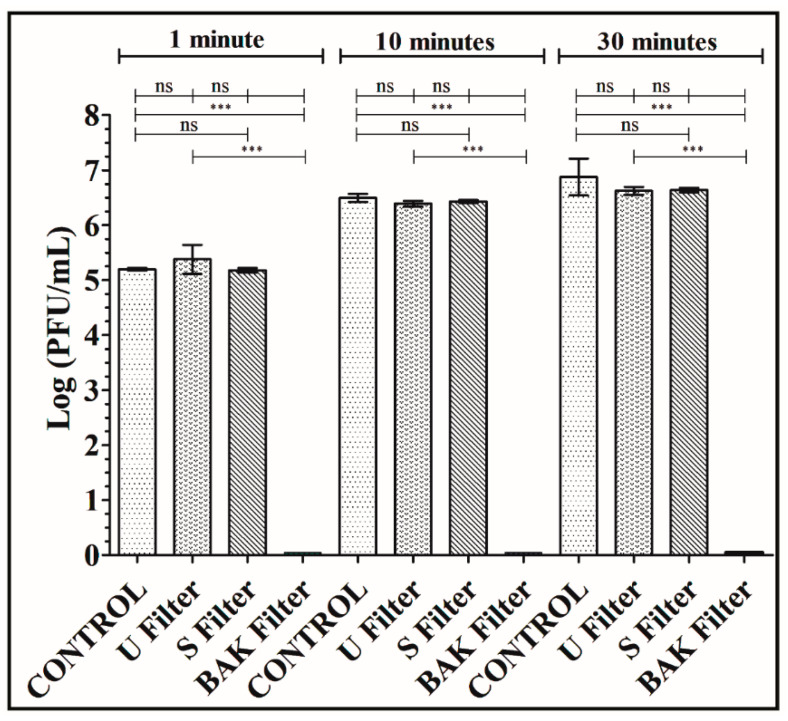
Titration after double-layer method with the phage phi 6 viral model. Logarithm of plaque-forming units per mL (log(PFU/mL)) of the control, untreated filter (U filter), filter treated by dip coating with the ethanol-based solvent (S filter) and filter with the biofunctional BAK coating (BAK filter) at 1, 10 and 30 min of viral contact.

Phage phi 6 is a three-part, segmented, double-stranded RNA virus totaling ~13.5 kb in length. Even though this type of lytic bacteriophage belongs to group III of the Baltimore classification [30], it was proposed here as a viral model of SARS-CoV-2, due to biosafety reasons, as it also has a lipid membrane around its nucleocapsid. Thus, the BAK Filter showed potent antiviral activity (100% of viral inhibition, see Figure 3 and Figure 4). Bacterial lawns had clearly grown in the plate and no plaques were observed after 1, 10 or 30 min of contact between the BAK filter and the SARS-CoV-2 viral model. Furthermore, the U filter and S filter showed similar results to control of no antiviral activity (see Figure 3 and Figure 4).

The phage titers of each type of face mask filter sample were calculated and compared with the control (see Figure 4).

Figure 4 shows that the titers obtained by contacting the phages with the U or S filter are similar to the control. However, the BAK filter displayed a strong phage inactivation.

**Figure 5:**
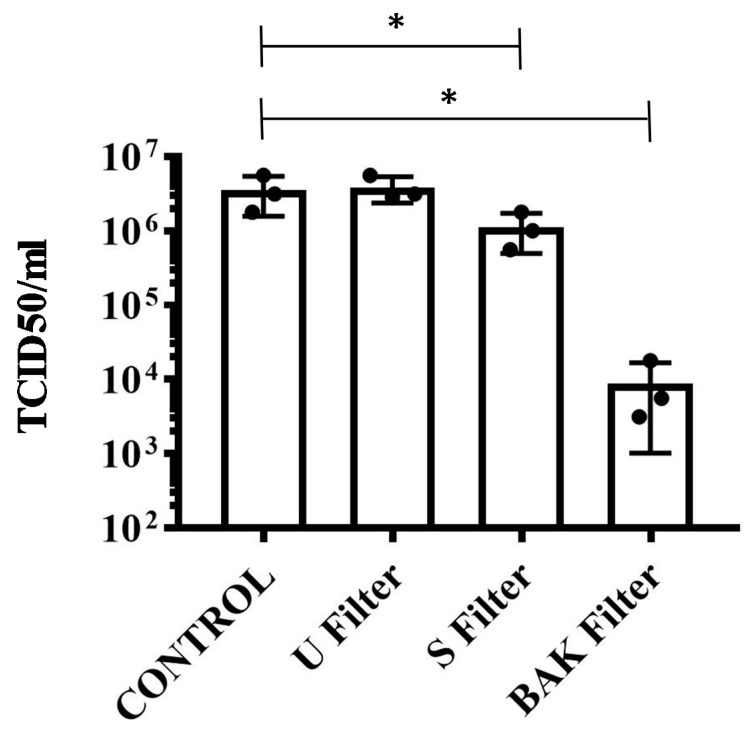
Reduction of infectious titers of SARS-CoV-2 after 1 min of contact. Untreated filter (U filter), filter treated with the ethanol solvent (S filter), filter with the biofunctional BAK coating (BAK filter) and control via the TCID50/mL method. A dot plot is data set based on the value of each point.

The results achieved with the TCID50/mL method about the reduction of infectious titers of SARS-CoV-2 after 1 min of contact with the control, the U filter, the S filter and the BAK filter containing the biofunctional coating are shown in Figure 5.

These results clearly demonstrate that the BAK filter is very effective against SARS-CoV-2 even after 1 min of contact. This is also in good agreement with the antiviral results of the biosafe viral model used in this study (see Figure 3 and Figure 4). The reduction of infection titers in PFU/mL determined by the double-layer assay for the phage phi 6 and by the TCID50/mL method for SARS-CoV-2 is shown in Table 1.

### 3.4. Antibacterial Tests

**Figure 6:**
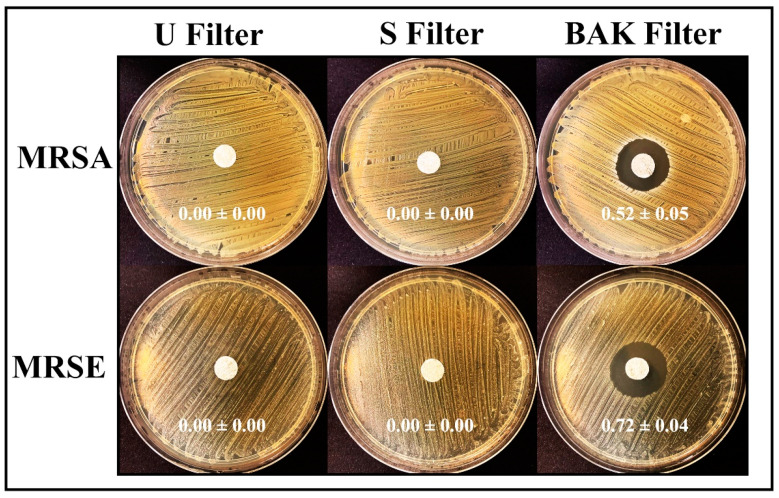
Antibacterial agar disk diffusion tests. Untreated filter (U filter), filter treated by dip coating with the ethanol-based solvent (S filter) and filter with the biofunctional BAK coating (BAK filter) after 24 h of culture at 37 °C. The normalized widths of the antibacterial *halos*, expressed as mean ± standard deviation and calculated with Equation (1), are shown in each image.

The antibacterial results of the treated and untreated filters against MRSA and MRSE multidrug-resistant bacteria are shown in Figure 6.

The filter treated by dip coating with 70% ethyl alcohol containing 0.1% benzalkonium chloride showed high antibacterial activity against MRSA and MRSE, being even more effective against the last strain. The antimicrobial mode of action of quaternary ammonium compounds (QACs) such as BAK against both bacterial and viral phospholipid membranes is attributed to the positively charged nitrogen atoms. This causes eradication of bacteria and common viruses such as influenza by disrupting their phospholipid bilayer membrane [32], the glycoproteinaceous envelope and the associated spike glycoproteins interacting with the ACE2 receptor in the infection of host cells [55]. For this reason, BAK is extensively found in many household disinfecting wipes and sprays and is also used as an additive in various soaps and non-alcohol-based hand sanitizers [31,56,57].

Here in this paper, a new face mask filter with antiviral and antibacterial properties against Gram-positive multidrug-resistant bacteria to reduce COVID-19 infections (from touching the filter masks and aerosol transmission in both senses) is demonstrated. This face mask filter has been developed here by dip coating (a simple, low-cost, reliable and reproducible method) a commercial non-woven filter where a thin coating of BAK was deposited onto the surface by physical adsorption [58]. The same biofunctional coating procedure could be applied to any type of face mask or biodegradable filters. This also represents a solution to the need for bio-based facemasks to counter coronavirus outbreaks [42]. The manufacturing procedure by dip coating with BAK opens up a broad range of applications that urgently demand new antimicrobial approaches. Thus, this technology may also be used for the fabrication of antimicrobial clothes, gloves, etc., for health personnel or to produce antimicrobial filters able to inactivate aerosols containing SARS-CoV-2 or Gram-positive multidrug-resistant bacteria in other applications. The antibacterial activity of this type of filter against MRSA and MRSE, and their viral inhibition capacity against SARS-CoV-2 as well as the enveloped phage phi 6 viral model demonstrates their broad antipathogenic protection.

## 4. Conclusions

This is the first report of the development of a face mask filter capable of inactivating SARS-CoV-2 and multidrug-resistant bacteria such as methicillin-resistant *Staphylococcus aureus* and *Staphylococcus epidermidis*, a new promising tool to combat the increasing COVID-19 spread. This antimicrobial non-woven face mask filter was produced by a reproducible and economic procedure using benzalkonium chloride that provides excellent antiviral properties against SARS-CoV-2 (>99% of viral inhibition after 1 min of contact) and the phage phi 6 (100% of viral inhibition after 1 min of contact), which was used here as a biosafe viral model of SARS-CoV-2. Therefore, the developed antiviral filter can be used in face masks and other protective tools, and thus is very promising to prevent the spread of SARS-CoV-2. Nonetheless, further research is required in order to ensure the safe use of the developed filters in the present COVID-19 pandemic.

## Figures and Tables

**Table 1:** Reduction of infection titers determined by the double layer assay for the phage phi 6 and via the TCID50/mL method for SARS-CoV-2. Control, untreated filter (U filter), filter treated with the ethanol solvent (S filter), filter with the biofunctional BAK coating (BAK filter).

Sample	Phi 6 at 1 min (PFU/mL)	Phi 6 at 10 min (PFU/mL)	Phi 6 at 30 min (PFU/mL)	SARS-CoV-2 at 1 min (PFU/mL)
Control	1.6 × 10^5^ ± 1.4 × 10^4^	3.2 × 10^6^ ± 8.9 × 10^5^	1.4 × 10^7^ ± 1.8 × 10^7^	3.5 × 10^6^ ± 1.9 × 10^6^
U Filter	3.5 × 10^5^ ± 3.9 × 10^5^	2.5 × 10^6^ ± 5.1 × 10^5^	4.3 × 10^6^ ± 1.3 × 10^6^	3.9 × 10^6^ ± 1.5 × 10^6^
S Filter	1.5 × 10^5^ ± 2.4 × 10^4^	2.7 × 10^6^ ± 2.8 × 10^5^	4.4 × 10^6^ ± 7.4 × 10^5^	1.1 × 10^6^ ± 6.2 × 10^5^
BAK Filter	0.0 ± 0.0	0.0 ± 0.0	0.0 ± 0.0	8.9 × 10^3^ ± 7.8 × 10^3^

## Data Availability

Data are contained within the article.
